# Clinical study to evaluate the efficacy and safety of home-used LED and IRED mask for crow’s feet: A multi-center, randomized, double-blind, sham-controlled study

**DOI:** 10.1097/MD.0000000000041596

**Published:** 2025-02-14

**Authors:** Sang Hyun Park, Seong Oh Park, Jae-A Jung

**Affiliations:** a Department of Plastic and Reconstructive Surgery, Hanyang University College of Medicine, Seoul, Korea; b Department of Plastic and Reconstructive Surgery, Seoul National University Hospital, Seoul National University College of Medicine, Seoul, Korea; c Department of Plastic and Reconstructive Surgery, Hanyang University Guri Hospital, Hanyang University College of Medicine, Seoul, Korea.

**Keywords:** crow’s feet, facial rejuvenation, LED and IRED mask

## Abstract

**Background:**

As the elderly population continues to grow, the demand for antiaging products is increasing concurrently. On our face, wrinkles begin to form first around the eyes, where the skin is the thinnest. Previous studies have suggested that irradiating the skin with light-emitting diode (LED)/infrared emitting diode (IRED) light at 600 to 660 nm/800 to 860 nm, stimulates the cells of the dermis and epidermal tissue and is effective in wrinkle improvement and antiaging. Therefore, we aimed to evaluate the efficacy and safety of low-level light therapy masks.

**Materials and methods:**

A randomized, sham device-controlled, double-blind clinical trial was conducted at 2 institutions. Sixty Asian descent individuals between the ages of 30 and 65 years who showed type II to V skin type on the Fitzpatrick scale were included. Among participants with a score of 2 to 4 on the crow’s feet grading scale (CFGS) at rest (without expression), those who sought temporary improvement in both crow’s feet were selected. The participants were categorized into 2 groups: the experimental group, which used a device with a combination of 630 nm LED (max 10 mW/cm^3^) and 850 nm IRED (max 10 mW/cm^3^), and the control group, which used the sham device. Efficacy evaluation included various evaluations, including the CFGS as rated by independent raters, CFGS scores assigned by investigators, and the Global Aesthetic Improvement Scale evaluation by both investigators and the participants.

**Results:**

After using the LED mask for 16 weeks, the CFGS score of the independent raters and investigators showed significant differences at 8, 12, and 16 weeks. In addition, considering the success criteria of this study, a comparison of independent raters showed an improvement rate of ≥69.2% (full analysis set [FAS]: 86.2%, per-protocol set [PPS]: 89.3%) and a difference of ≥49.2% from the control group (FAS: 69.5%, PPS: 72.6%). The change in scores from the baseline showed significant differences between the test group and the control groups at 8, 12, and 16 weeks for both independent raters and investigators.

**Conclusion:**

LED and IRED phototherapies at 630 nm and 850 nm, respectively, are effective, safe, well-tolerated, and painless treatment for skin rejuvenation.

## 1. Introduction

Light therapy has long been used to treat various diseases. The use of sunlight to treat skin diseases was practiced thousands of years ago in Egypt, India, and China. Treatment using sunlight was reexamined by Nobel Prize winner Niels Ryberg Finsen in 1903 which later became the beginning of phototherapy using artificial light.^[1]^

A treatment method using a light-emitting diode (LED) light source for treating skin diseases has been developed recently.^[2]^ An LED light source can effectively treat a wide portion of the diseased area with an appropriate light output, unlike a high-power laser, which intensively treats a localized area. The LED light source has a narrow wavelength line and emits a light source of a specific wavelength; thus, harmful ultraviolet (UV) rays or infrared (IR) rays are not emitted, and there are few side effects. In addition, because it does not damage skin tissue or eyes owing to its low energy, the United States Food and Drug Administration (US FDA) has approved treatment using LED light sources in the visible and near-IR range for human use. The physical characteristics of the LED light source include a long lifespan, low power consumption, environmental friendliness, and a small volume, making it easy to utilize and store in a small space.

Phototherapy, described as low-level light therapy (LLLT), is based on the absorption of photons from an LED light source by chromophores or photo acceptors in cellular tissues and the stimulation of cellular metabolic activity. The light absorbed by the cell increases the synthesis of reactive oxygen species (ROS) and adenosine triphosphate (ATP) in the cell tissues. In addition, cells exposed to red and near-IR light release nitric oxide (NO). ROS influence gene expression. Light of a particular wavelength at an appropriate time and period is absorbed by photoreceptors in cells, such as cytochrome c oxidase. It photo-degrades inhibitory NO, promoting enzyme activation, mitochondrial metabolism, and ATP production. Consequently, proteins such as hemoglobin and myoglobin release additional NO, leading to a series of intracellular biochemical reactions.^[3]^

Research and development of LED therapy devices for skin disease treatment began in the mid-2000s, which is relatively recent compared to the laser therapy devices being manufactured by Photo Therapeutics Ltd (Altrincham, UK) since 1998. Several clinical studies have confirmed that irradiating the skin with LED/ infrared emitting diode (IRED) light at 600 to 660 nm/800 to 860 nm stimulates the cells of the dermis and epidermal tissue, effectively improving wrinkles and antiaging. In addition, light in the 600 to 650 nm and 800 to 860 nm bands increased fibroblasts in human dermis and increased the synthesis of collagen and elastin proteins.^[4]^ Recently, Li et al confirmed that collagen synthesis and elastin synthesis increased even when low-intensity light of 640 and 830 nm (0.5 mW/cm^2^) (0.3 J) was used for 10 minutes every day.^[5]^ In other words, the energy of LED light helps skin elasticity and pore contraction, preventing wrinkles.

In this study, the efficacy and safety of temporary improvement of wrinkles around the eyes by stimulating the dermis layer on the face with LED and IRED light of 630 nm and 850 nm, respectively, were compared with those of the sham device.

## 2. Materials and methods

### 2.1. Study design

This study was designed as a randomized, sham device-controlled, double-blind clinical trial conducted at 2 different institutions. Sixty candidates voluntarily agreed to participate in the clinical trial and informed consent was obtained from the patient for publication of this case report details. The selected cases were randomized into the experimental and control groups that used an experimental device and a sham device, respectively, for 12 weeks. This study was conducted in accordance with the World Medical Association Declaration of Helsinki and the protocol was approved by the Institutional Review Board of our University Medical Center (2022-07-018).

### 2.2. Patient enrollment

This study included individuals of Asian descent between the ages of 30 and 65 years who showed type II to V skin type on the Fitzpatrick scale. Among individuals with a score of 2 to 4 on the crow’s feet grading scale (CFGS) at rest (without expression), those who sought temporary improvement in both crow’s feet were selected. The principal investigators at both institutions enrolled the participants. The selected patients were required to discontinue all dermatological treatments, including facial wrinkle improvement, during the clinical trial period. We excluded patients who had received cosmetic treatment such as laser, light treatment, and surgery on the face within the last 6 months, or had received filler treatment using collagen, hyaluronic acid filler, or other materials. Patients with skin diseases or wound infections at the site of application of the clinical trial medical device were also excluded. The demographic data of the participants are described in Tables 1 and 2.

**Table 1:** Participant demographics in the study.

	Total	
	N	%
Screening and acquiring informed consent	60	100.00
Participation in the study	59	98.33
Completion status	59	
Completed	58	98.31
Early termination	1	1.69
Reasons for early termination and dropouts	2	
Withdrawal of consent due to adverse events	2	3.33

**Table 2:** Demographic characteristics of the participants.

		Experimental group (N = 29)				Control group (N = 30)				*P*-value
		N		%		N		%		
Sex	Male	2		6.9		2		6.7		1.000*
	Female	27		93.1		28		93.3		
Selected site	Right	16		55.2		13		43.3		.439*
	Left	3		44.8		17		56.7		
		Mean	SD	Median	IQR	Mean	SD	Median	IQR	
Age		46.07	5.87	46.00	25	48.37	7.55	49.00	26	.195**

IQR = interquartile range, SD = standard deviation.

* Fisher exact test.

** Independent *t*-test.

### 2.3. Randomization and intervention (experimental and sham devices)

**Figure 1.**
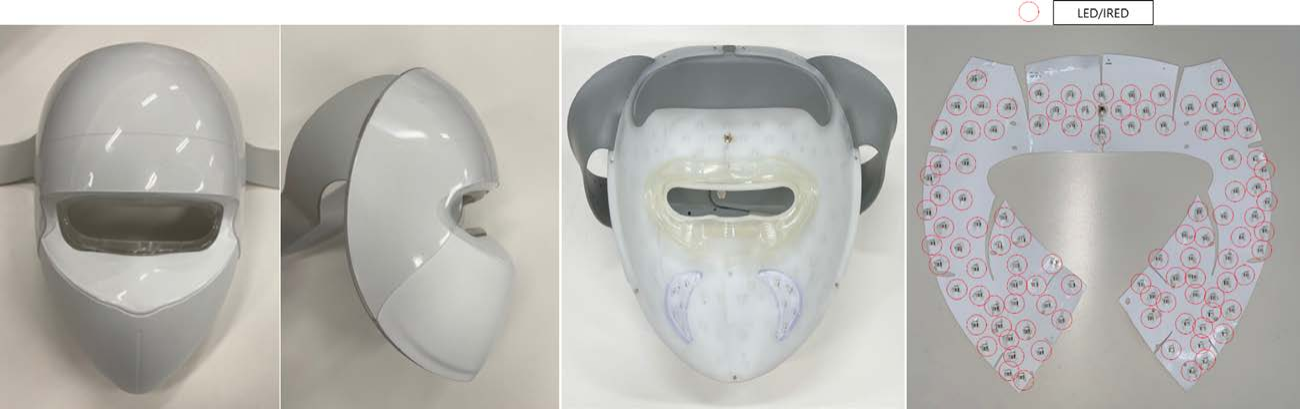
Crow’s feet grading scale (CFGS) and global anesthetic improvement scale (GAIS).

The Medical Statistics Consulting Department randomized the selected cases into experimental and control groups by generating a random sequence using a complete randomization method (PASS 16, NCSS, LLC., Kaysville). Each group used the experimental device or a sham device that was contained in a set of sealed boxes, only recorded with a serial number in the order of registration. Both participants and clinical investigators remained blinded throughout the study. Participants were secretly divided into 2 groups; 1 used the experimental device while the other used the sham helmet-type devices at home (Fig. 1). The experimental group used a device containing a combination of a medical laser device and a low-level light irradiation device, composed of a 630 nm LED (max 10 mW/cm^2^) and 850 nm IRED (max 10 mW/cm^2^) (Easy Claire; Y&J Bio, Seoul, Korea). The sham device had the same appearance and components as the outline of experimental devices for clinical trials, but did not emit at IR (850 nm), and its LED (630 nm) output was at one-tenth of the original intensity, while the method of use was the same. Both groups applied the device to their face, including the area being measured, for 9 min, 5 times a week for a total of 12 weeks (total: 60 times and 540 minutes).

### 2.4. Efficacy evaluation and patient satisfaction

**Figure 2.**
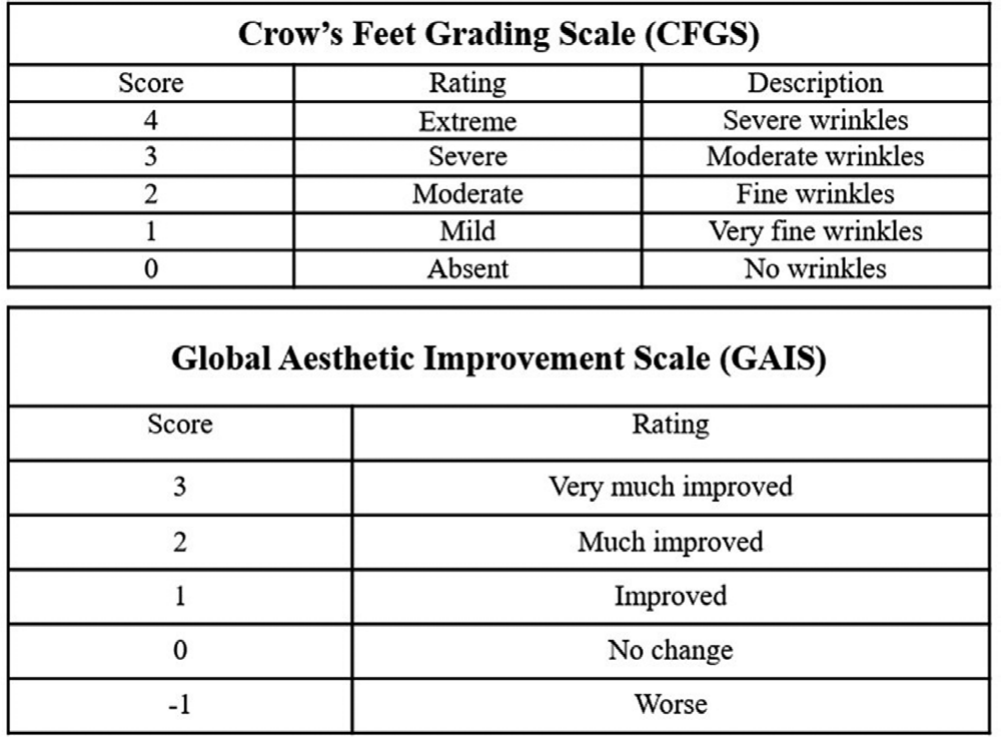
The experimental group was exposed to a test device emitting light at wavelengths of 630 nm for the LED and 850 nm for the IRED, with an output of max 10 mW/cm^2^ ± 20% for both. The control group was treated with a sham device, identical in appearance to the test device. However, the sham device did not emit at IR (850 nm), and its LED (630 nm) output was reduced to one-tenth of the original intensity. IRED = infrared emitting diode, LED = light-emitting diode.

Participants visited at 4, 8, and 12 weeks during the clinical trial medical device application period, including screening and baseline visits, with a maximum of 6 visits at 16 weeks, including a follow-up visit. Efficacy and safety were evaluated for 4 weeks after 12 weeks of application of the clinical trial medical device. Efficacy evaluation included assessments of the CFGS by both an independent rater and the investigator, along with global aesthetic improvement scale (GAIS) evaluations conducted by the investigator and the participant (Fig. 2). Safety evaluation included identification of adverse events, vital signs, and physical examinations. In addition, at every visit for efficacy evaluation, including the baseline visit, a photo was taken of the area where the clinical trial medical device was applied, and an independent evaluator conducted the CFGS evaluation using the captured photo (EOS M6 Mark 2, Canon, Japan).

### 2.5. Statistical analysis

Primary efficacy was evaluated by 3 independent evaluators 12 weeks after the first application of the clinical trial devices, which presented the frequency and percentage of subjects whose wrinkles around the eyes improved. Comparative evaluation of the test group and control groups was performed using Pearson chi-square test, Fisher exact test or *Z*-test using SPSS software (version 20.0, SPSS Inc., Chicago). The secondary efficacy evaluation was performed by comparing the groups using Pearson chi-square test, Fisher exact test, or *Z*-test. In addition, the significance of the differences between the groups in the evaluation variables at each time point was tested using McNemar test or McNemar exact test. For continuous evaluation variables, the mean, standard deviation (SD), median, and interquartile range (IQR) at each time point are presented for each application group. Evaluation variables were compared between the application groups using the independent 2-sample *t*-test or Mann–Whitney *U* test according to normality at each time point and compared with an analysis of covariance (ANCOVA) using the general linear model after correcting the baseline. In addition, the significance within the group of the change in the evaluation variable from baseline, at each time point was tested using repeated-measures analysis of variance (ANOVA) and paired *t*-test or Wilcoxon signed-rank test according to normality. Mean differences were considered to be significant at *P* < .05. Results are shown as the mean ± SD.

## 3. Results

Sixty participants, comprising 4 men and 56 women, participated in the trial at the 2 institutions. Regarding the sex of the participants, the test group had 2 males (7.1%) and 26 females (92.9%), while the control group had 2 males (6.7%) and 28 females (93.3%). One female participant discontinued for personal reasons after the baseline visit and was withdrawn from the trial; a total of 59 participants were included in the final analysis of the results. The average age was 46.07 years in the experimental group and 48.37 years in the sham device group (*P* = .195). Selected sites were 16 right (57.1%) and 12 left (42.9%) in the test group, along with 13 right (43.3%) and 17 left (56.7%) in the control group. There was no significant difference in the *P*-value between the test and control groups (Tables 1 and 2).

### 3.1. Primary efficacy evaluation

The temporary improvement in wrinkles around the eyes was evaluated by an independent evaluator 12 weeks after the first application of the medical device for clinical trials, and the ratio of those with improved wrinkles around the eyes was compared between the test and control groups. It was defined as ‘improved wrinkles around the eyes’ when the CFGS score at rest (without expression) decreased by ≥1 point compared to that before application of the medical device among participants who were treated with clinical trial medical devices. Twelve weeks after the initial application of the investigational medical device, the frequency and percentage (%) of participants showing improvement in periorbital wrinkles were evaluated by an independent evaluator; 25 of the 29 (86.2%) patients in the full analysis set (FAS) group and 5 of 30 in the control group (16.7%) showed improvement. In the per-protocol set (PPS) group, 25 out of 28 (89.3%) patients in the test group and 5 of 30 (16.7%) in the control group showed improvement (Table 3). An independent rater compared the 2 groups at 12 weeks after the initial application of the investigational device to compare the temporal improvement of wrinkles around the eyes between the test and the control groups using Fisher exact test. The test results showed that the difference between the test group and the control group was significant (FAS: *P* = .000, PPS: *P* = .000), and the effectiveness of the LED mask for the temporary improvement of wrinkles around the eyes was confirmed. In addition, the McNemar test was conducted for within-group comparisons at 12 weeks compared to the baseline. There was a significant difference in the test group (FAS: *P* = .000, PPS: *P* = .000); however, no significant difference was observed in the control group (FAS: *P* = .125, PPS: *P* = .125).

**Table 3:** Comparison of the proportion of the participants with improvement in crow’s feet wrinkles as assessed by independent evaluator at week 12.

		Experimental group		Control group		*P*-value
		N	%	N	%	
FAS (N = 59)	No change, worsening	4	13.8	25	83.3	.000*
	Improvement	25	86.2	5	16.7	
	*P*-value	.000**		.125**		
PPS (N = 58)	No change, worsening	3	10.7	25	83.3	.000*
	Improvement	25	89.3	5	16.7	
	*P*-value	.000**		.125**		

FAS = full analysis set, PPS = per-protocol set.

* Fisher exact test.

** 4 wk vs 12 wk McNemar test.

### 3.2. Secondary efficacy evaluation

#### 3.2.1. *Proportion of subjects with improved crow’s feet wrinkles (independent rater*)

**Figure 3.**
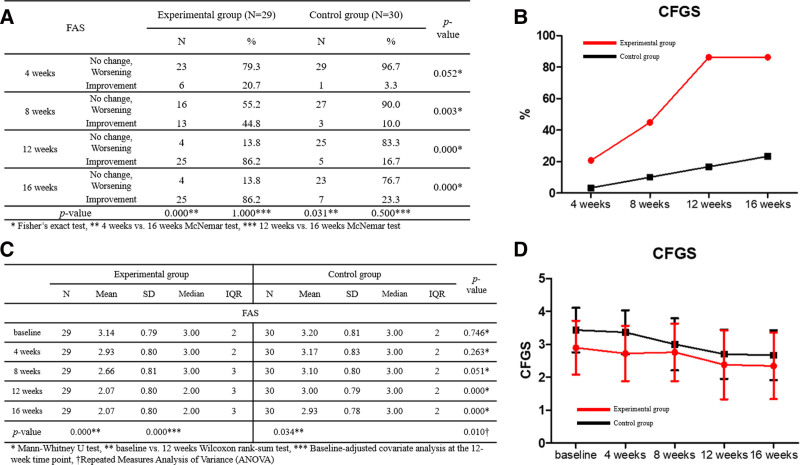
(A) The proportion of participants with improved crow’s feet wrinkles, as assessed by an independent evaluator. (B) Comparison of participants’ proportions. (C) Comparison of crow’s feet grading scale (CFGS) scores. (D) Changes in scores.

The percentage of participants with improvement in CFGS, as assessed by an independent rater, was compared between the test and control groups using Fisher exact test. FAS showed no significant difference between the 2 groups at 4 weeks, but the differences were significant at 8, 12, and 16 weeks (*P* = .052, .003, .000, and .000, respectively). In PPS, a significant difference between the 2 groups was confirmed at 4, 8, 12, and 16 weeks (*P* = .048, .003, .000, and .000, respectively) (Fig. 3). In addition, the McNemar test was conducted for intragroup comparison at 16 weeks vs 4 weeks, and significant differences were confirmed in both the test and control groups (FAS: *P* = .000 vs .031, PPS: *P* = .000 vs .031). Comparison of the CFGS scores evaluated by independent raters between the test group and the control group using baseline-corrected 12-week covariance analysis showed a significant difference between the 2 groups (FAS: *P* = .000, PPS: *P* = .000).

#### 3.2.2. *Proportion of subjects with improved crow’s feet wrinkles (investigator*)

A comparison of the ratio of subjects with improved periorbital wrinkles, as evaluated by the investigator, between the test and control groups, using Fisher exact test showed that the difference between the 2 groups was not significant at 4 weeks after the first application of the clinical trial medical device (*P* = .532). However, after the eighth week, the difference between the 2 groups was significant (*P* = .001, .001, and .002, respectively). The PPS group showed similar results as the FAS group, and the *P*-values at 4, 8, 12, and 16 weeks were .336, .003, .001, and .003, respectively.

**Figure 4.**
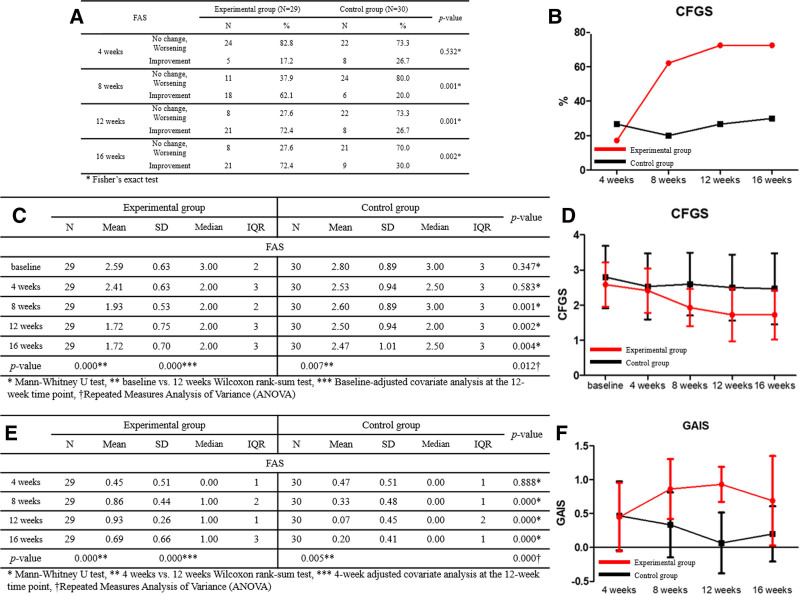
(A) Proportion of participants with investigator-assessed improvement in crow’s feet wrinkles. (B) Comparison of participants’ proportions. (C) Comparison of crow’s feet grading scale (CFGS) scores. (D) Changes in scores. (E) Comparison of global aesthetic improvement scale (GAIS) scores. (F) Changes in scores.

A comparison of the test and control groups using covariate analysis at 12 weeks, correcting the GAIS score for the 4th week, confirmed a significant difference between the 2 groups (FAS: *P* = .000, PPS: *P* = .000). In addition, analysis using repeated-measure ANOVA showed a significant difference (FAS: *P* = .000, PPS: *P* = .000) in the change in scores between the 2 groups at 4, 8, 12, and 16 weeks. A comparison between the test group and the control group using the Mann–Whitney *U* test, showed that the difference between the 2 groups was not significant at 4 weeks after the first application of the clinical trial device (FAS: *P* = .888, PPS: *P* = .773), but was significant after 8 weeks (*P* = .000, .000, and .000 at 8, 12, and 16 weeks, respectively, Fig. 4).

### 3.3. Comparison of investigator-assessed GAIS scores

A comparison of the test and control groups using covariate analysis at 12 weeks, correcting the GAIS score for the 4th week, showed a significant difference between the 2 groups (FAS: *P* = .000, PPS: *P* = .000). In addition, analysis using repeated-measures ANOVA showed a significant difference in the change in scores between the 2 groups at 4, 8, 12, and 16 weeks (FAS: *P* = .000, PPS: *P* = .000). A comparison between the test and control groups using the Mann–Whitney *U* test revealed that the difference between the 2 groups was not significant at 4 weeks after the first application of clinical trial medical devices (FAS: *P* = .888, PPS: *P* = .773), but was significant after the 8th week (FAS: *P* = .000, .000, .000, PPS: *P* = .000, .000, .001). Additionally, a Wilcoxon rank-sum test was conducted to compare the groups at weeks 4 and 12, and significant differences were confirmed in both the test and control groups (FAS: *P* = .000 and .005, respectively, PPS: *P* = .000 and .005, respectively, Fig. 4).

### 3.4. Comparison of subject-assessed GAIS scores

**Figure 5.**
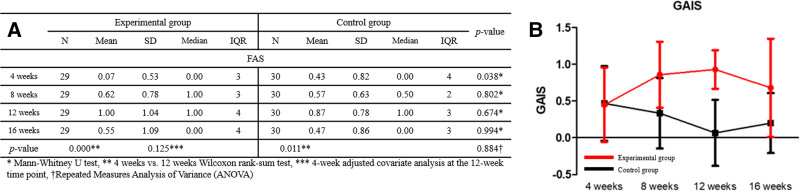
(A) Comparison of global aesthetic improvement scale (GAIS) scores, as assessed by the participant. (B) Changes in scores.

**Figure 6.**
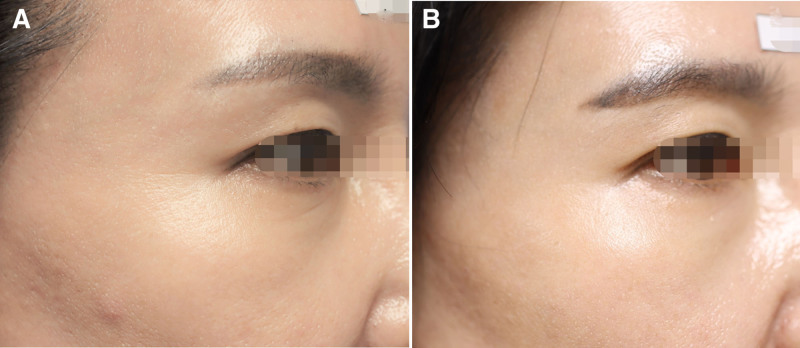
A 53-year-old female, (A) with baseline score of 2 points on the left as assessed by an independent evaluator using Crow’s Feet Grading Scale (CFGS), which (B) improved to 1 point after 16 wk of device application.

**Figure 7.**
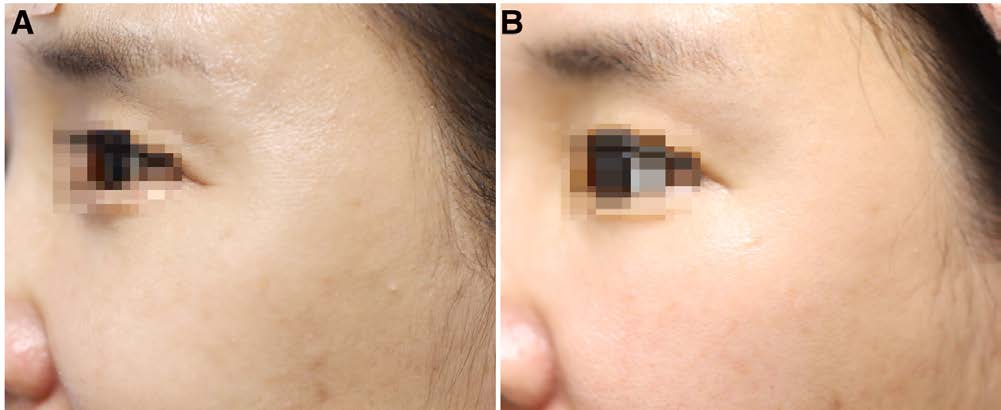
A 43-year-old female, (A) with baseline score of 3 points on the left as assessed by an independent evaluator using Crow’s Feet Grading Scale (CFGS), which (B) improved to 1 point after 16 wk of device application.

**Figure 8.**
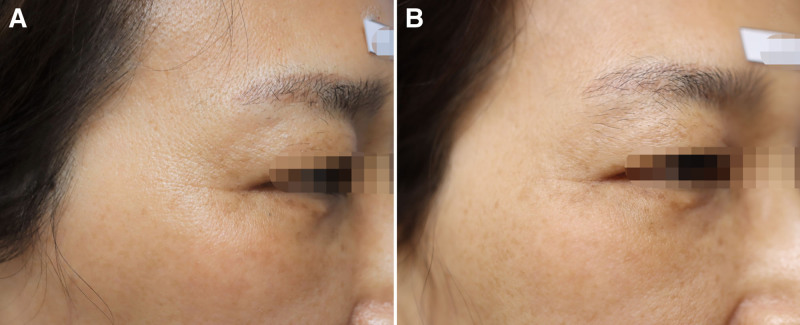
A 53-year-old female, (A) with baseline score of 3 points on the right as assessed by an independent evaluator using crow’s feet grading scale (CFGS), which (B) improved to 1 point after 16 wk of device application.

As a result of comparing the test and control groups using covariate analysis at 12 weeks, corrected for the 4th week GAIS score for the eye area as evaluated by the participant, no significant difference was observed between the 2 groups (FAS: *P* = .125, PPS: *P* = .094). In addition, analysis using repeated-measures ANOVA showed no differences in the change in scores between the 2 groups at 4, 8, 12, and 16 weeks (FAS: *P* = .884, PPS: *P* = .827). Accordingly, a Wilcoxon rank-sum test was conducted to compare the groups at weeks 4 and 12, and significant differences were confirmed in both the test and control groups (FAS: *P* = .000 and .011, respectively, PPS: *P* = .000 and .011, respectively) (Fig. 5). Photographs of patients showing clinical improvement are provided in Figures 6 to 8.

### 3.5. Adverse events

Of the 60 participants in this study, 4 (6.67%) reported adverse events. Dry skin occurred in 1 patient in the test group, therefore, the patient was withdrawn, and it was confirmed that no sequelae remained. In addition, coronavirus infection occurred in 1 patient in the test group and allergic conjunctivitis occurred in 1 patient. In the control group, 1 case of mushroom allergy occurred, but there were no serious adverse reactions. Thus, the safety of the medical device in clinical trials was confirmed.

## 4. Discussion

Skin is the organ most sensitive to hormones in the human body. Keratin cells, Langerhans cells, melanocytes, sebaceous glands, and fibroblasts present in the skin are affected by hormones, and when estrogen levels decrease, the capillaries in the skin decrease. In addition, collagen, elastin, and hyaluronic acid, which are the 3 main components of skin tissue, decrease with aging. The epidermis thins, forming wrinkles and reducing skin stiffness. There are various types of wrinkles on the face due to the loss of elasticity of the skin caused by aging and various environmental factors.

Wrinkles occur first around the eyes, where the skin is the thinnest, followed by the forehead, nasolabial folds under the chin, and ears; it can be corrected through surgery and injection therapy, which is also widely used. Furthermore, as the number of consumers unable to access regular care due to time, location, or economic reasons increases, the demand for home care appliances is rapidly increasing.^[6]^

Over the past few years, the use of LED home devices has attracted considerable interest in the antiaging market. These LLLT devices are sources of photo-biomodulation therapy that have been used not only to promote wound healing, but also to produce anti-inflammatory effects and photo-rejuvenation, and for treating various dermatological disorders.^[7–9]^ Among the wavelengths, red LEDs have been shown to increase collagen type 1 and matrix metalloproteinase-9 expression, which allows the degradation of fragmented collagen and leads to neo-collagen production.^[4]^

No serious adverse events occurred during the study period. Four cases of minor events occurred, but there was no significant association to this clinical study. Rather, approximately a quarter of the participants noticed skin-whitening effect of the therapy. Some participants reported that their skin tone became brighter, and in some the blemishes faded. Both participants and evaluators recognized the effect of brightening skin tone in the test group. However, objective research on this topic is not included in this study.

The present study had several limitations. One limitation of this study was the short follow-up period. Furthermore, the possibility that patients may have received other skin care services during the clinical trial cannot be excluded, although it was officially forbidden. During the clinical trial period, participants were prohibited from using whitening agents or wrinkle-improving functional cosmetics or undergoing Botox injections, laser treatments, and filler injections. Additional long-term follow-up studies should be conducted to more accurately evaluate the results and side effects. Furthermore, future research on the scientific and quantitative whitening effects of the LED masks is essential.

## 5. Conclusion

In this study, following the use of LED mask and sham device for 16 weeks, the CFGS assessment of independent raters and investigators showed significant differences at 8, 12, and 16 weeks. In addition, the comparison of independent raters indicated that the study met its success criteria, demonstrating an improved rate of 69.2% (FAS: 86.2%, PPS: 89.3%) or more and a difference from the control group of 49.2% (FAS: 69.5%, PPS: 72.6%) or more were confirmed. The change in scores from the baseline showed significant differences between the test and control groups at 8, 12, and 16 weeks in FAS and PPS for both independent assessors and testers.

Thus, LED and IRED phototherapies at 630 nm and 850 nm, respectively, are effective, safe, well-tolerated, and painless treatment for skin rejuvenation. We recommend the use of a combination of these 2 wavelengths of light to maximize the effect by utilizing the advantages specific to each wavelength.

## Acknowledgments

This study was funded by Y&J Bio, Seoul, Republic of Korea. Study research and design, data collection, data analysis, interpretation of results, and writing of the manuscript were independent of funding sources. The authors have no significant commercial interests and have no conflicts of interest to disclose. Additionally, we state that this study was presented at the 2023 APS Conference.

## Author contributions

**Data curation:** Sang Hyun Park.

**Investigation:** Seong Oh Park.

**Project administration:** Jae-A Jung.

**Supervision:** Jae-A Jung.

**Writing – review & editing:** Seong Oh Park.

**Writing – original draft:** Jae-A Jung.

## References

[1] Roelandts R. A new light on Niels Finsen, a century after his nobel prize. Photodermatol Photoimmunol Photomed. 2005;21:115–7. 15888126 10.1111/j.1600-0781.2005.00160.x

[2] Barolet D. Light-Emitting Diodes(LEDs) in dermatology. Semin Cutan Med Surg. 2008;27:227–38. 19150294 10.1016/j.sder.2008.08.003

[3] Hamblin MR Demidova TN. Mechanisms for low-light therapy. Proc SPIE. 2006;6140:1–12.

[4] Russell BA Kellett N Reilly LR. A study to determine the efficacy of combination LED light therapy (633 nm and 830 nm) in facial skin rejuvenation. J Cosmet Laser Ther. 2005;7:196–200. 16414908 10.1080/14764170500370059

[5] Li WH Seo I Kim B Fassih A Southall MD Parsa R. Low-level red plus near infrared lights combination induces expressions of collagen and elastin in human skin in vitro. Int J Cosmet Sci. 2021;43:311–20. 33594706 10.1111/ics.12698

[6] Ng JNC Wanitphakdeedecha R Yan C. Efficacy of home-use light-emitting diode device at 637 and 854-nm for facial rejuvenation: a split-face pilot study. J Cosmet Dermatol. 2020;19:2288–94. 32649063 10.1111/jocd.13613

[7] Yoon JS Ku WY Lee JH Ahn HC. Low-level light therapy using a helmet-type device for the treatment of androgenetic alopecia: a 16-week, multicenter, randomized, double-blind, sham device-controlled trial. Medicine (Baltimore). 2020;99:e21181. 32702878 10.1097/MD.0000000000021181 PMC7373546

[8] Gavish L Houreld NN. Therapeutic efficacy of home-use photobiomodulation devices: a systematic literature review. Photobiomodul Photomed Laser Surg. 2019;37:4–16. 31050938 10.1089/photob.2018.4512

[9] Elsaie ML Choudhary S Leiva A Nouri K. Nonablative radiofrequency for skin rejuvenation. Dermatol Surg. 2010;36:577–89. 20384760 10.1111/j.1524-4725.2010.01510.x

